# The genome sequence of the Pine Cone moth, *Dioryctria mendacella* (Staudinger, 1859) (Lepidoptera: Pyralidae)

**DOI:** 10.12688/wellcomeopenres.27800.1

**Published:** 2026-09-14

**Authors:** oreina, Papillons de France, Arun Arumugaperumal, Charlotte J. Wright, Joana I. Meier, Mark L. Blaxter, Wellcome Sanger Institute Tree of Life Management, Samples and Laboratory team, Wellcome Sanger Institute Scientific Operations: Sequencing Operations, Wellcome Sanger Institute Tree of Life Core Informatics team, Tree of Life Core Informatics collective, Project Psyche Community

**Affiliations:** 1 Oreina, Thoury-Férottes (Seine-et-Marne), France; 2 Department of Biotechnology, Rajalakshmi Engineering College, Thandalam, Chennai, Tamilnadu, India; 3 Tree of Life, Wellcome Sanger Institute, Hinxton, England, UK

**Keywords:** Dioryctria mendacella, Pine Cone moth, genome sequence, chromosomal, Lepidoptera

## Abstract

We present a genome assembly from a female specimen of *Dioryctria mendacella* (Arthropoda; Insecta; Lepidoptera; Pyralidae). The assembly contains two haplotypes with total lengths of 801.36 megabases and 732.65 megabases. Most of the haplotype 1 assembly (99.75%) was scaffolded into 32 chromosomal pseudomolecules, including the W and Z sex chromosomes. Haplotype 2 was assembled to scaffold level. The mitochondrial genome has also been assembled, with a length of 15.39 kilobases. Gene annotation of this assembly by Ensembl identified 15,868 protein-coding genes. This work is part of Project Psyche, a collaborative programme generating genomes for European butterflies and moths.

## Species taxonomy

Eukaryota; Opisthokonta; Metazoa; Eumetazoa; Bilateria; Protostomia; Ecdysozoa; Panarthropoda; Arthropoda; Mandibulata; Pancrustacea; Altocrustacea; Allotriocarida; Hexapoda; Insecta; Dicondylia; Pterygota; Neoptera; Eumetabola; Endopterygota; Aparaglossata; Panorpida; Amphiesmenoptera; Lepidoptera; Glossata; Neolepidoptera; Heteroneura; Ditrysia; Obtectomera; Pyraloidea; Pyralidae; Phycitinae; *Dioryctria*; *Dioryctria mendacella* (Staudinger, 1859) (NCBI:txid500707)

## Background

The Pine Cone moth, *Dioryctria mendacella*, is a significant pest of Mediterranean pines, particularly the stone pine ( *Pinus pinea* L.). Its wingspan is 22–27 mm. The labial palpi, flanking the proboscis, are long, slender, curved, and pointed at the apex. The antennae are filiform in females, while in males, pectination is short with a few scales appressed at the base of the flagellum. The forewing is moderately broad with a blunt apex, light ash grey in colour, with whitish transverse lines edged with black; the antemedial line is edged further towards the inner side, the postmedial line is weakly angled, and the median area has a grey discal spot and a large blackish spot touching the inner margin. The hindwing is light grey ( Leraut, 2014).

*Dioryctria mendacella* has been recorded in Portugal, Spain, France, Greece, Akrotiri and Dhekelia, Israel, Italy, Morocco, Croatia, Russia, Lebanon, Ukraine and Algeria ( GBIF Secretariat, 2023; Leraut, 2014). The host plants include *Pinus halepensis* Mill., *P. pinaster* Aiton, *P. pinea*, and *P. brutia* Ten. ( Hall *et al.*, 2017; Leraut, 2014). Its life cycle includes at least two generations per year, with females laying eggs on the cones. The larvae bore into the cones, causing deformations, abnormal growth, and cone abortion, resulting in substantial seed loss. They also attack pine shoots, leading to shoot dieback and wilting ( Calama *et al.*, 2017).

Larvae are purplish-red dorsally and whitish-pink ventrally, with a shiny black head and shield. They feed inside the cone, causing asymmetric development and creating visible holes in the cones that can be seen from the outside. These holes, along with brown spots on immature green cones, can be used to identify infested cones. Infestation of stone pine cones by this species can be identified by brown spots on green cones. The infestation can be detected on the outside of the cone through visible holes. As it progresses, the holes are filled with resin and larval excreta ( European Institute of Planted Forest (IEFC), 2018). The Mediterranean stone pine, *Pinus pinea*, exhibits a masting habit, which is affected by moth infestation. Reduced seed production also threatens the next generation of trees. The pattern and extent of insect attack on the trees remain unpredictable. Studies using pheromone traps to attract the moth and study its seasonal flight patterns have been conducted ( Hall *et al.*, 2017). In Portugal, the moth’s flight period was observed from February to early December, with nocturnal flight activity ( Naves *et al.*, 2023). The level of infestation depends on site and environmental conditions. During winter, the larvae hibernate inside the cone; therefore, increased temperatures favour winter survival and indirectly lead to greater pine cone damage ( Calama *et al.*, 2017). The edible nuts of the stone pine have high commercial value. The pest can be controlled by setting traps with sex pheromones ( Naves *et al.*, 2023).

Sequencing the genome of this pest may help in developing new molecular technologies to mitigate the devastating effects of infestation.

## Methods

### Sample acquisition

The specimen used for genome sequencing was an adult female *Dioryctria mendacella* (specimen ID SAN28000415, ToLID ilDioMend1; Figure 1), collected from Saint-Étienne-de-Baïgorry, Pyrénées-Atlantiques, Pays Basque, France (latitude 43.1814, longitude −1.3739) on 2024-05-19. The specimen was collected and identified by members of *oreina*, Papillons de France.

**Figure 1.**
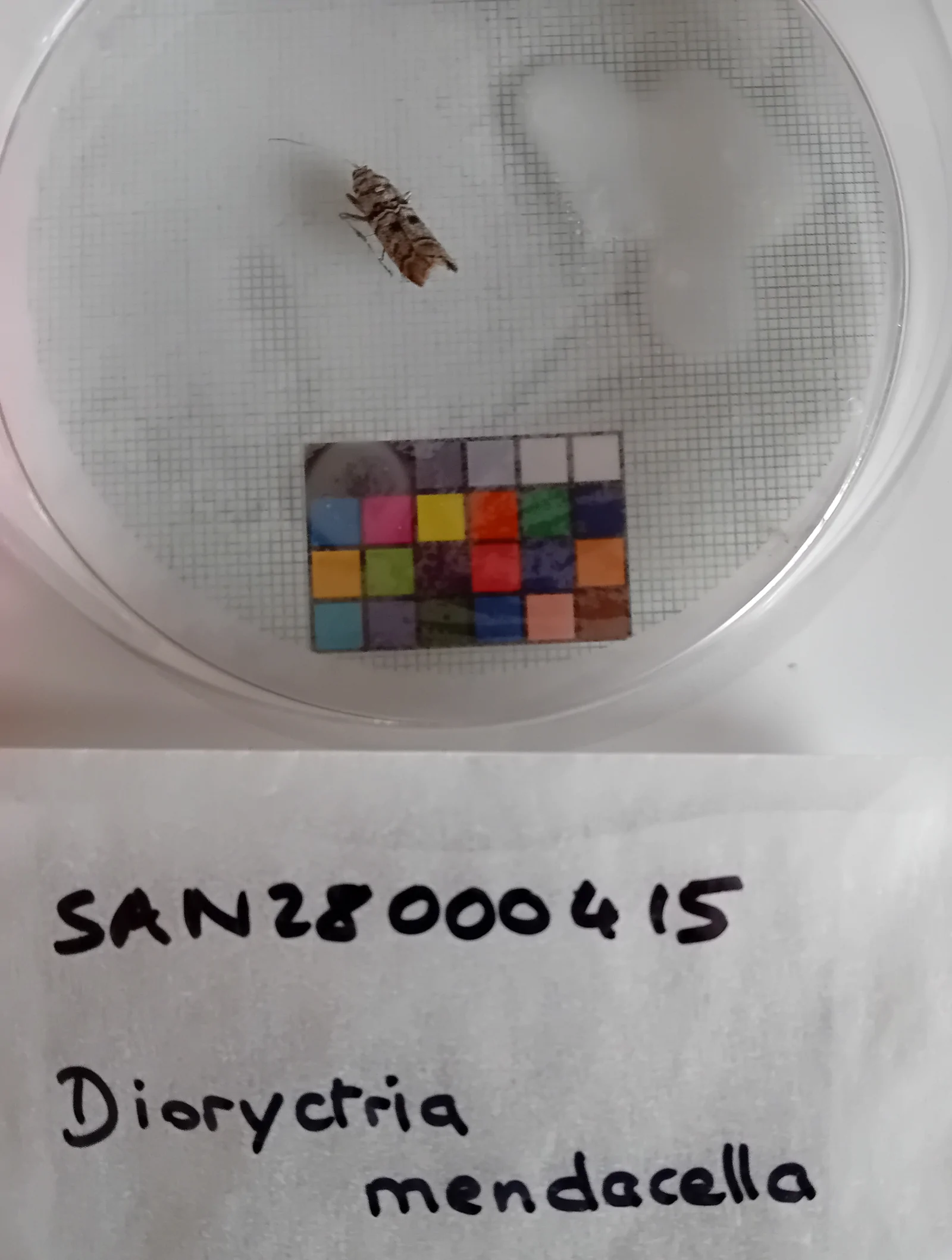
Voucher photograph of the *Dioryctria mendacella* (ilDioMend1) specimen used for genome sequencing.

### Nucleic acid extraction

Protocols for high molecular weight (HMW) DNA extraction developed at the Wellcome Sanger Institute (WSI) Tree of Life Core Laboratory are available on protocols.io ( Howard *et al.*, 2025). For HMW DNA extraction, tissue from the thorax of ilDioMend1 was homogenised by powermashing using a PowerMasher II tissue disruptor. HMW DNA was extracted in the WSI Scientific Operations core using the Automated MagAttract v2 protocol. DNA was sheared into an average fragment size of 12–20 kb following the Megaruptor®3 for LI PacBio protocol. Sheared DNA was purified by automated SPRI (solid-phase reversible immobilisation). The concentration of the sheared and purified DNA was assessed using a Nanodrop spectrophotometer and Qubit Fluorometer using the Qubit dsDNA High Sensitivity Assay kit. Fragment size distribution was evaluated by running the sample on the Femto Pulse system. For this sample, the final post-shearing DNA had a Qubit concentration of 50.3 ng/μL and a yield of 2,364.10 ng, with a fragment size of 15.4 kb. The 260/280 spectrophotometric ratio was 1.94, and the 260/230 ratio was 2.26. The Genomic Quality Number (GQN) was 6.4.

### PacBio HiFi library preparation and sequencing

Library preparation and sequencing took place in the WSI Scientific Operations core. Library PSYCHE15123096 was prepared with the SMRTbell Prep Kit 3.0 (Pacific Biosciences) according to the manufacturer’s instructions. The kit includes reagents for end repair/A-tailing, adapter ligation, post-ligation SMRTbell bead clean-up, and nuclease treatment. Size selection and clean-up were performed using diluted AMPure PB beads (Pacific Biosciences). DNA concentration was quantified with a Qubit Fluorometer v4.0 (Thermo Fisher Scientific) and the Qubit 1X dsDNA HS assay kit. Final library fragment size was assessed with the Agilent Femto Pulse Automated Pulsed Field CE Instrument (Agilent Technologies) using the gDNA 55 kb BAC analysis kit.

The library was sequenced on a Revio instrument (Pacific Biosciences, California, USA). The prepared libraries selected for multiplexing were pooled based on genome size, library molarity and the intended plex level. Primers were annealed and polymerases were bound to generate circularised complexes following the manufacturer’s instructions. Complexes were purified using SMRTbell beads, diluted to the Revio loading concentration, and spiked with a Revio sequencing internal control. The pooled libraries were sequenced on a Revio 25M Plex SMRT cell in a 2-plex run. SMRT Link software (Pacific Biosciences) was used to configure and monitor the run and to perform primary and secondary data analysis.

### Hi-C

***Sample preparation and crosslinking***

Hi-C data were generated from the ilDioMend1 head sample using the Arima-HiC v2 kit (Arima Genomics). Following the manufacturer’s instructions, tissue was fixed and DNA crosslinked using TC buffer to a final formaldehyde concentration of 2%. The tissue was homogenised using the Diagnocine Power Masher-II. Crosslinked DNA was digested with a restriction enzyme master mix, biotinylated, and ligated. Clean-up was performed with SPRISelect beads before library preparation. DNA concentration was measured with the Qubit Fluorometer (Thermo Fisher Scientific) and Qubit HS Assay Kit. The biotinylation percentage was estimated using the Arima-HiC v2 QC beads.

***Hi-C library preparation and sequencing***

Biotinylated DNA constructs were fragmented using a Covaris E220 sonicator and size-selected to 400–600 bp using SPRISelect beads. DNA was enriched with Arima-HiC v2 kit Enrichment beads. End repair, A-tailing, and adapter ligation were carried out with the NEBNext Ultra II DNA Library Prep Kit (New England Biolabs), following a modified protocol where library preparation occurs while DNA remains bound to the Enrichment beads. Libraries were amplified with KAPA HiFi HotStart mix and a custom Unique Dual Index (UDI) barcode set (Integrated DNA Technologies). Depending on sample concentration and biotinylation percentage determined at the crosslinking stage, libraries were amplified with 10–16 PCR cycles. Post-PCR clean-up was performed with SPRISelect beads. Libraries were quantified using the AccuClear Ultra High Sensitivity dsDNA Standards Assay Kit (Biotium) and a FLUOstar Omega plate reader (BMG Labtech).

Prior to sequencing, libraries were normalised to 10 ng/μL. Normalised libraries were quantified again to create equimolar and/or weighted 2.8 nM pools. Pool concentrations were checked using the Agilent 4200 TapeStation (Agilent) with High Sensitivity D500 reagents before sequencing. Libraries were sequenced on the Illumina NovaSeq X, generating paired-end 150-bp reads.

### Genome assembly

Prior to assembly of the PacBio HiFi reads, a database of *k*-mer counts ( *k* = 31) was generated from the filtered reads using FastK. The *k*-mer frequency distribution was analysed with GenomeScope (version 2.1.0) ( Ranallo-Benavidez *et al.*, 2020), providing fitted point estimates of genome size, heterozygosity and repeat content.

The HiFi reads were assembled using Hifiasm in Hi-C phasing mode ( Cheng *et al.*, 2021, 2022), producing two haplotypes. Hi-C reads ( Rao *et al.* , 2014) were mapped to contigs using bwa-mem2 ( Vasimuddin *et al.*, 2019). Contigs were further scaffolded with Hi-C data in YaHS ( Zhou *et al.*, 2023), using the --break option for handling potential misassemblies. The scaffolded assemblies were evaluated using Gfastats ( Formenti *et al.* , 2022), BUSCO ( Manni *et al.*, 2021) and MerquryFK ( Rhie *et al.*, 2020).

The mitochondrial genome was assembled using MitoHiFi ( Uliano-Silva *et al.* , 2023), which runs MitoFinder ( Allio *et al.*, 2020) and uses these annotations to select the final mitochondrial contig and to ensure the general quality of the sequence.

### Assembly curation

The assembly was decontaminated using the Assembly Screen for Cobionts and Contaminants ( ASCC) pipeline. The flat files and maps for curation were generated with TreeVal. Manual curation was conducted primarily in PretextView and HiGlass ( Kerpedjiev *et al.* , 2018). Scaffolds were visually inspected and corrected as described by Howe *et al*. (2021). The two haplotype assemblies were combined for curation. Manual corrections included three breaks and 34 joins. This reduced the scaffold count by 18.4%, increased the scaffold N50 by 2.1%, and increased the total assembly length by 0.9%. The curation process is described at sanger-tol/curation-resources. A Hi-C contact map of the haplotype 1 assembly was generated with PretextSnapshot.

### Assembly quality assessment

The MerquryFK tool ( Rhie *et al.*, 2020), run in a Singularity container ( Kurtzer *et al.*, 2017), was used to evaluate *k*-mer completeness and assembly quality for both haplotypes using the *k*-mer databases ( *k* = 31) computed prior to genome assembly. The analysis outputs included assembly QV scores and completeness statistics.

The haplotype 1 assembly was assessed using BlobToolKit ( Challis *et al.*, 2020). PacBio reads were mapped to the haplotype 1 assembly to derive sequence coverage, and BUSCO ( Manni *et al.*, 2021) was used to assess gene-set completeness. DIAMOND BLASTp searches of extracted BUSCO proteins and DIAMOND BLASTx searches of assembly sequences against UniProt Reference Proteomes ( Buchfink *et al.*, 2021), followed by BLASTn searches of sequences without DIAMOND BLASTx hits against the NCBI nt database ( Altschul *et al.*, 1990), supported taxonomic assignment. Coverage, sequence composition, taxonomic assignment and BUSCO results were integrated into a BlobDir for interactive inspection and visualisation. The snail and blob plots were rendered locally from this hosted BlobDir using BlobTk v0.8.3. The scaffold-length radial scale function for the snail plot was set to linear ( Challis & Blaxter, 2026). The blob plot used circular markers with area proportional to scaffold length ( Challis *et al.*, 2020).

We used *busco-alg-painter* v0.1.0, a Python reimplementation and extension of *lep_busco_painter*, to paint Merian elements along chromosomes. Merian elements represent the 32 ancestral linkage groups reconstructed for Lepidoptera ( Wright *et al.*, 2024). The BUSCO-to-Merian reference assignments were derived from *lep_busco_painter*. Complete single-copy and duplicated BUSCO genes from the lepidoptera_odb10 results were assigned to Merian elements. Chromosome identifiers and lengths were obtained from NCBI Datasets, and BUSCO positions were plotted on chromosomes drawn to scale.

## Genome sequence report

### Sequence data

PacBio sequencing of the *D. mendacella* specimen generated 38.83 Gb (gigabases) from 4.18 million reads. We assembled the genome from these reads. GenomeScope analysis using a *p* = 2 model gave a *k*-mer-based haploid genome size estimate of 774.06 Mb, with heterozygosity of 0.70% and repeat content of 36.67%; the full-model fit was 97.87% (Figure 2). The GenomeScope estimates guided expectations for the assembly. The sequencing data provided approximately 49× coverage of the haplotype 1 assembly. Hi-C sequencing produced 130.98 Gb from 433.71 million reads, which were used to scaffold the haplotype 1 assembly. Table 1 summarises the specimen and sequencing details.

**Figure 2.**
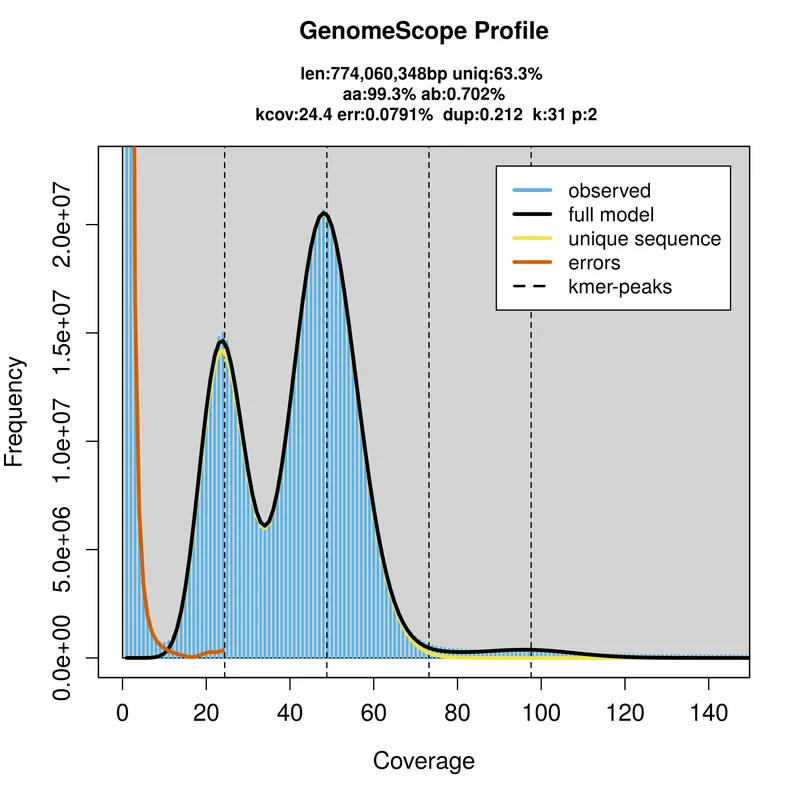
GenomeScope model fitted to the *k*-mer frequency distribution. The plot shows the observed and modelled *k*-mer spectra used to estimate genome size, heterozygosity and repeat content from unassembled sequencing reads.

**Table 1.** Specimen and sequencing data for *D. mendacella* (BioProject PRJEB82270)

**Platform**	**PacBio HiFi**	**Hi-C**
**ToLID**	ilDioMend1	ilDioMend1
**Specimen ID**	SAN28000415	SAN28000415
**BioSample (source individual)**	SAMEA115948970	SAMEA115948970
**BioSample (tissue)**	SAMEA115949482	SAMEA115949480
**Tissue**	thorax	head
**Instrument**	Revio (1 run)	Illumina NovaSeq X
**Library ID & run accession(s)**	PSYCHE15123096: ERR13946496	ERR13947524
**Read count total**	4.18 million reads	433.71 million read pairs
**Base count total**	38.83 Gb	130.98 Gb

### Assembly statistics

The genome was assembled into two haplotypes using Hi-C phasing. The haplotype 1 assembly was curated to chromosome level, while haplotype 2 was assembled to scaffold level. The haplotype 1 assembly, which is proposed as the reference assembly for this species, has a total length of 801.36 Mb in 79 scaffolds (excluding organelle sequences), with 113 gaps, and a scaffold N50 of 27.93 Mb (Table 2).

**Table 2.** Genome assembly statistics for *D. mendacella*

**Genome assembly**	**Haplotype 1**	**Haplotype 2**
**Assembly name**	ilDioMend1.hap1.1	ilDioMend1.hap2.1
**Assembly accession**	GCA_964374295.1	GCA_964374265.1
**Assembly level**	chromosome	scaffold
**Span (Mb)**	801.36	732.65
**Number of chromosomes**	32	-
**Number of contigs**	192	163
**Contig N50 (Mb)**	12.54	10.53
**Number of scaffolds (excluding organelle sequences)**	79	70
**Scaffold N50 (Mb)**	27.93	27.45
**Scaffold auN (Mb)**	26.4	25.6
**Longest scaffold length (Mb)**	37.14	31.43
**Sex chromosomes**	W and Z	-
**Organelles**	Mitochondrial genome: 15.39 kb	-

Most of the haplotype 1 assembly sequence (99.75%) was assigned to 32 chromosome-level scaffolds, representing 30 autosomes and the W and Z sex chromosomes. These chromosome-level scaffolds, confirmed by Hi-C data, are named according to size (Figure 3; Table 3). Chromosome painting of the haplotype 1 assembly with Merian elements illustrates the distribution of orthologues along chromosomes and highlights patterns of chromosomal evolution relative to Lepidopteran ancestral linkage groups (Figure 4). The Z and W chromosomes were assigned by copy number in the diploid genome and the Hi-C signal.

The mitochondrial genome was also assembled (length 15.39 kb, OZ206491.1). This sequence is included as a contig in the multifasta file of the haplotype 1 genome submission and as a standalone record.

**Figure 3.**
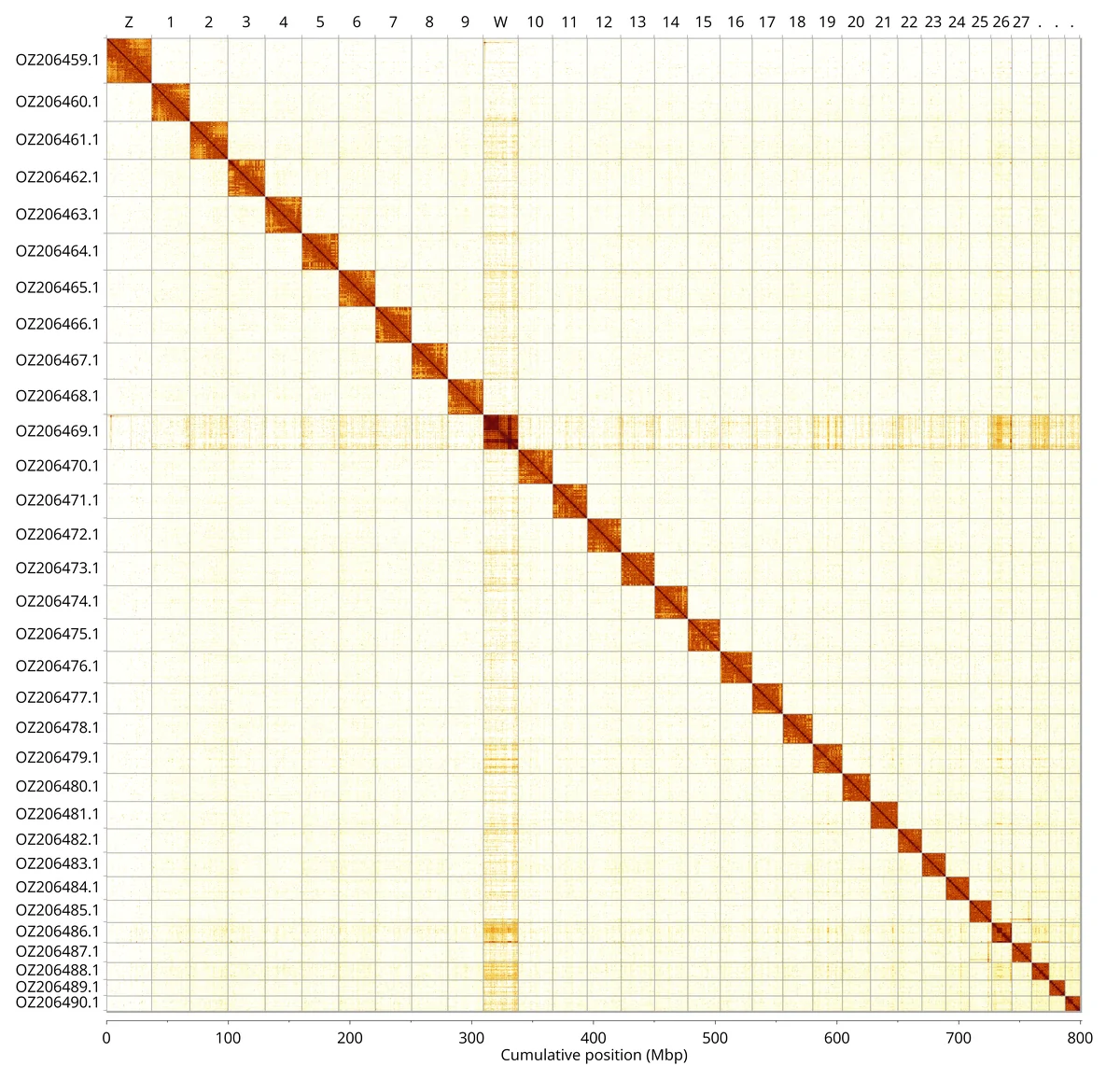
Hi-C contact map of the *D. mendacella* haplotype 1 assembly. Assembled chromosomes are shown in order of size and labelled along the axes. The plot was generated using PretextSnapshot.

**Table 3.** Chromosomal pseudomolecules in the haplotype 1 genome assembly of *D. mendacella* ilDioMend1

**INSDC accession**	**Molecule**	**Length (Mb)**	**GC%**	**Assigned Merian elements**
OZ206460.1	1	31.46	37	M2
OZ206461.1	2	31.03	37.50	M5
OZ206462.1	3	30.65	37.50	M17; M20
OZ206463.1	4	30.26	37.50	M1
OZ206464.1	5	30.15	37	M8
OZ206465.1	6	30	37.50	M7
OZ206466.1	7	29.86	37	M9
OZ206467.1	8	29.64	37	M12
OZ206468.1	9	29.13	37	M3
OZ206470.1	10	28.35	37	M18
OZ206471.1	11	28.17	37	M16
OZ206472.1	12	27.93	37	M6
OZ206473.1	13	27.35	37	M22
OZ206474.1	14	27.28	37.50	M4
OZ206475.1	15	26.60	37	M21
OZ206476.1	16	26.30	37	M15
OZ206477.1	17	25.11	37.50	M10
OZ206478.1	18	24.70	37.50	M11
OZ206479.1	19	24.36	37.50	M23
OZ206480.1	20	23.08	38	M14
OZ206481.1	21	22.30	37.50	M13
OZ206482.1	22	19.80	38	M24
OZ206483.1	23	19.53	38	M26
OZ206484.1	24	19.32	38	M19
OZ206485.1	25	18.33	38	M28
OZ206486.1	26	16.80	39	M30
OZ206487.1	27	16.07	38.50	M27
OZ206488.1	28	14.52	38.50	M31
OZ206489.1	29	13.06	38.50	M25
OZ206490.1	30	12.34	38.50	M29
OZ206469.1	W	28.77	37	-
OZ206459.1	Z	37.14	37	MZ

**Figure 4.**
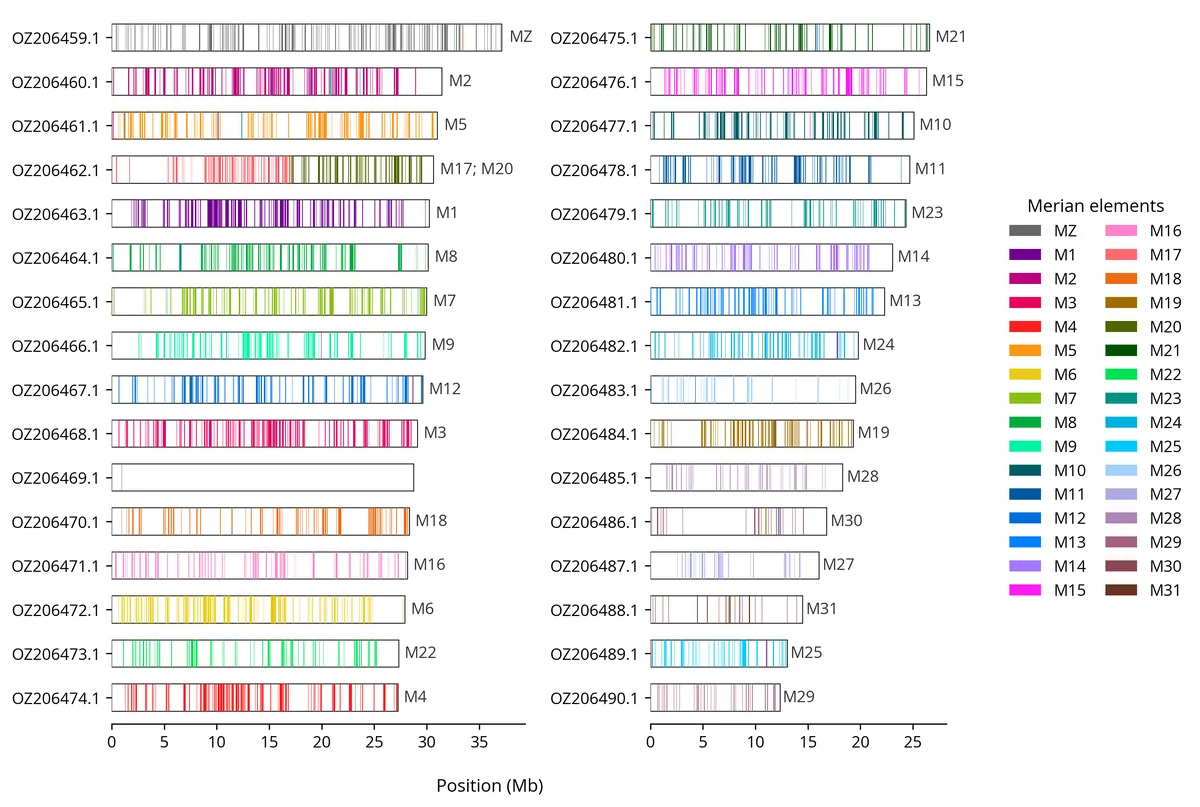
Merian elements painted across chromosomes in the ilDioMend1.hap1.1 haplotype 1 assembly of *D. mendacella*. Chromosomes are drawn to scale, with the positions of orthologues shown as coloured bars. Each orthologue is coloured by the Merian element that it belongs to. All orthologues which could be assigned to Merian elements are shown.

### Assembly quality metrics

For the haplotype 1 assembly, the estimated QV is 65.7, and for the haplotype 2 assembly, 66.0. When the two haplotypes are combined, the assembly achieves an estimated QV of 65.8. The *k*-mer completeness is 85.96% for the haplotype 1 assembly, 81.15% for the haplotype 2 assembly, and 99.37% for the combined haplotypes (Figure 5).

**Figure 5.**
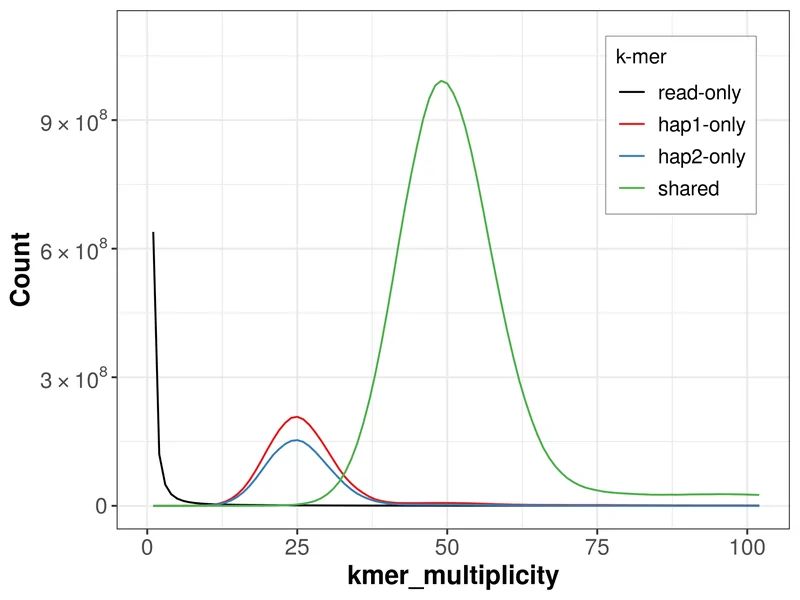
Evaluation of *k*-mer completeness using MerquryFK. This plot illustrates the recovery of *k*‐mers from the original read data in the haplotype 1 assembly, the haplotype 2 assembly and the combined haplotypes. The horizontal axis represents *k*‐mer multiplicity, and the vertical axis shows the number of *k*‐mers. The black curve represents *k*‐mers that appear in the reads but are not assembled. The green curve corresponds to *k*‐mers shared by both haplotypes, and the red and blue curves show *k*‐mers found only in one of the haplotypes.

BUSCO analysis using the lepidoptera_odb10 reference set ( *n* = 5,286) identified 99.0% of the expected gene set (single = 98.6%, duplicated = 0.4%) in the haplotype 1 assembly. For the haplotype 2 assembly, BUSCO analysis identified 94.7% of the expected gene set (single = 94.2%, duplicated = 0.5%).

The haplotype 1 assembly has a scaffold auN of 26.4 Mb. The snail plot in Figure 6 provides a summary of assembly contiguity, cumulative scaffold count, sequence composition and BUSCO completeness for the haplotype 1 assembly ( Challis & Blaxter, 2026). The relative sizes of the longest scaffold, scaffold N50 and N90, together with the dark-grey scaffold-length distribution, show how much of the assembly is contained in long scaffolds. The outer composition track shows variation in GC, AT and N content, while the central track shows the cumulative scaffold count and the BUSCO inset summarises gene-set completeness.

The blob plot in Figure 7 displays scaffolds from the haplotype 1 assembly by GC proportion and base coverage. Marker area is proportional to scaffold length, and colour indicates taxonomic assignment ( Challis *et al.*, 2020).

**Figure 6.**
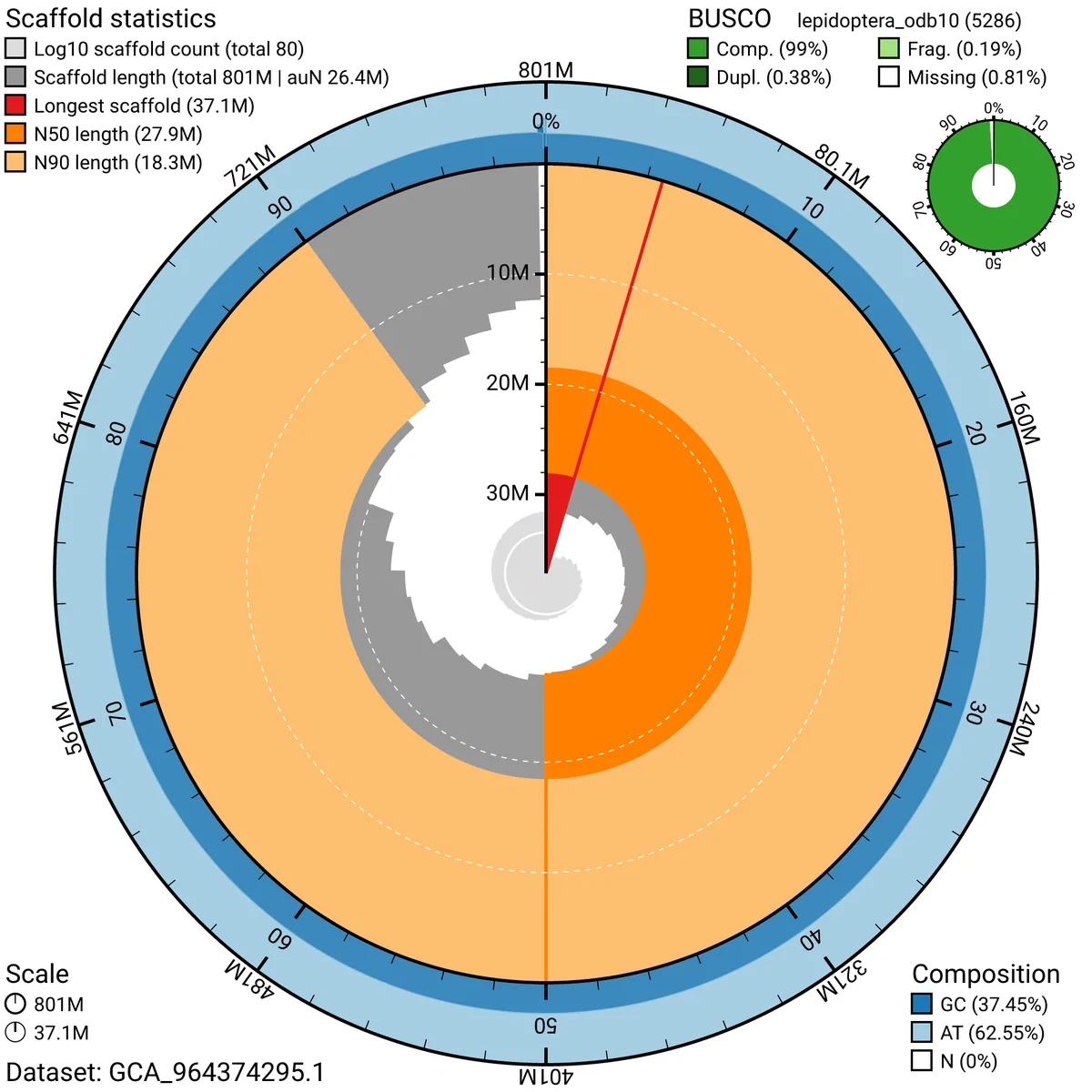
Assembly metrics for ilDioMend1.hap1.1, the haplotype 1 assembly. The BlobToolKit snail plot provides an overview of assembly metrics and BUSCO gene completeness. The circumference represents the length of the haplotype 1 assembly, and the main plot is divided into 1,000 bins around the circumference. The outermost blue tracks display the distribution of GC, AT, and N percentages across the bins. Scaffolds are arranged clockwise from longest to shortest and are depicted in dark grey. Scaffold length is shown on a linear radial scale, scaled to the longest scaffold. Red shading indicates the longest scaffold, and the deeper orange and pale orange shading represent the N50 and N90 lengths. The summary statistics include scaffold auN, the area under the scaffold Nx curve. The light grey spiral at the centre shows the cumulative scaffold count on a logarithmic scale. A summary of complete, fragmented, duplicated, and missing BUSCO genes in the lepidoptera_odb10 set is presented at the top right. The assembly can be explored interactively in the BlobToolKit viewer.

**Figure 7.**
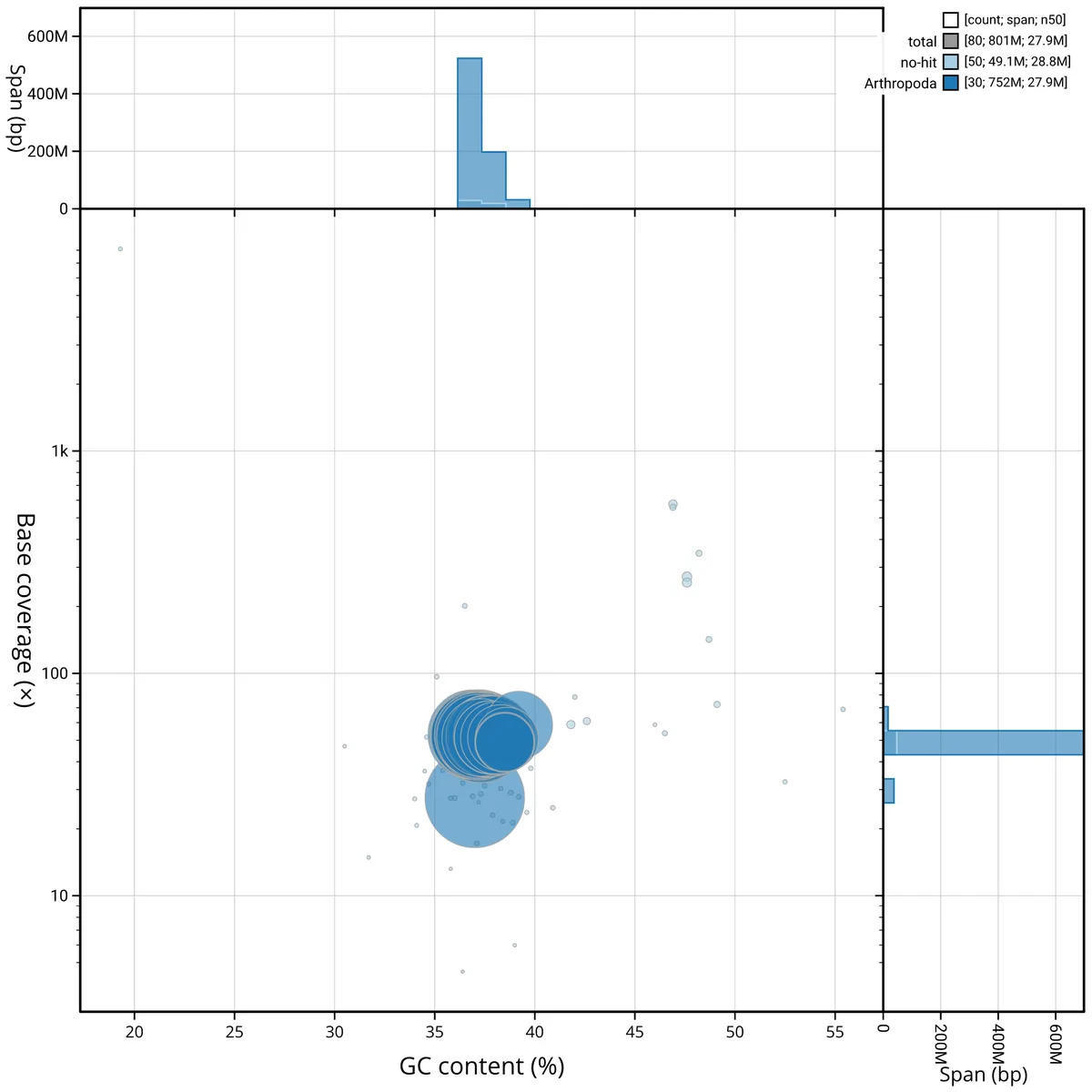
BlobToolKit blob plot for ilDioMend1.hap1.1, the haplotype 1 assembly. The plot shows base coverage (vertical axis) and GC content (horizontal axis). Circles represent scaffolds; marker area is proportional to scaffold length, and colour indicates taxonomic assignment. The histograms along the axes display the total length of sequences distributed across different levels of coverage and GC content. The assembly can be explored interactively in the BlobToolKit viewer.

Table 4 lists the assembly metric benchmarks adapted from Rhie *et al.*. (2021) the Earth BioGenome Project (EBP) Report on Assembly Standards. The three-part EBP metric, calculated for the haplotype 1 assembly, is **7.C.Q65**.

**Table 4.** Earth BioGenome Project summary metrics for the *D. mendacella* assembly

**Measure**	**Value**	***Benchmark***
EBP summary (haplotype 1)	7.C.Q65	*6.C.Q40*
Contig N50 length	12.54 Mb	*> 1 Mb*
Scaffold N50 length	27.93 Mb	*= chromosome N50*
Consensus quality (QV)	Haplotype 1: 65.7; haplotype 2: 66.0; combined: 65.8	*≥ 40*
*k*-mer completeness	Haplotype 1: 85.96%; Haplotype 2: 81.15%; combined: 99.37%	*> 90%*
BUSCO	Haplotype 1: C:99.0% [S:98.6%, D:0.4%], F:0.2%, M:0.8%, n:5,286; Haplotype 2: C:94.7% [S:94.2%, D:0.5%], F:0.2%, M:5.2%, n:5,286	*S > 90%; D < 5%*
Percentage of assembly assigned to chromosomes	99.75%	*≥ 90%*

**Notes:** The EBP summary uses log10(Contig N50); chromosome-level (C) or log10(Scaffold N50); Q (Merqury QV). BUSCO v5.5.0 using the lepidoptera_odb10 lineage dataset: C=complete; S=single-copy; D=duplicated; F=fragmented; M=missing; n=orthologues.

### Genome annotation report

The *D. mendacella* genome assembly (GCA_964374295.1) was annotated by Ensembl at the European Bioinformatics Institute (EBI). The annotation comprises 41,043 transcripts from 15,868 protein-coding genes and 9,742 non-coding genes. The average transcript length is 20,725.76 bp, with an average of 1.60 coding transcripts per gene and 6.55 exons per transcript. For further information, please refer to the Ensembl annotation page.

### Data availability

European Nucleotide Archive: Dioryctria mendacella. Accession number: PRJEB82270; URL: https://identifiers.org/ena.embl/PRJEB82270. The genome sequence is released openly for reuse. The *D. mendacella* genome sequencing initiative is part of the Sanger Institute Tree of Life Programme ( PRJEB43745) and Project Psyche ( PRJEB71705). All raw sequence data and the haplotype assemblies have been deposited in INSDC databases. Raw data and assembly accession identifiers are reported in Table 1 and Table 2.

Pipelines used for genome assembly at the WSI Tree of Life are available at https://pipelines.tol.sanger.ac.uk/pipelines. Table 5 lists software versions used in this study.

**Table 5.** Software versions and sources used for *D. mendacella*

**Software**	**Version**	**Source**
BLAST	2.14.0	https://ftp.ncbi.nlm.nih.gov/blast/executables/blast+/
BlobToolKit	4.3.9	https://github.com/genomehubs/blobtoolkit
BlobTk (hosted BlobDir)	0.5.1	https://github.com/genomehubs/blobtk
BlobTk (snail and blob plot renderer)	0.8.3	https://github.com/genomehubs/blobtk
BUSCO	5.5.0	https://gitlab.com/ezlab/busco
busco-alg-painter	0.1.0	https://github.com/Karenvn/busco-alg-painter
bwa-mem2	2.2.1	https://github.com/bwa-mem2/bwa-mem2
DIAMOND	2.1.8	https://github.com/bbuchfink/diamond
fasta_windows	0.2.4	https://github.com/tolkit/fasta_windows
FastK	1.2	https://github.com/thegenemyers/FASTK
GenomeScope	2.1.0	https://github.com/tbenavi1/genomescope2.0
Gfastats	1.3.6	https://github.com/vgl-hub/gfastats
Hifiasm	0.19.8-r603	https://github.com/chhylp123/hifiasm
HiGlass	1.13.4	https://github.com/higlass/higlass
MerquryFK	1.1.0-c1	https://github.com/thegenemyers/MERQURY.FK
Minimap2	2.24-r1122	https://github.com/lh3/minimap2
MitoHiFi	3.2.2	https://github.com/marcelauliano/MitoHiFi
MultiQC	1.14; 1.17 and 1.18	https://github.com/MultiQC/MultiQC
Nextflow	23.10.0	https://github.com/nextflow-io/nextflow
PretextSnapshot	0.0.6	https://github.com/sanger-tol/PretextSnapshot
PretextView	1.0.3	https://github.com/sanger-tol/PretextView
samtools	1.19.2	https://github.com/samtools/samtools
sanger-tol/ascc	0.1.0	https://github.com/sanger-tol/ascc
sanger-tol/blobtoolkit	0.6.0	https://github.com/sanger-tol/blobtoolkit
sanger-tol/curationpretext	1.4.2	https://github.com/sanger-tol/curationpretext
Seqtk	1.4-r122	https://github.com/lh3/seqtk
Singularity	3.9.0	https://github.com/sylabs/singularity
TreeVal	1.1.1	https://github.com/sanger-tol/treeval
YaHS	1.2a.2	https://github.com/c-zhou/yahs

### Author information

Contributors are listed at the following links:

- Members of *oreina* (Papillons de France)
- Members of the Wellcome Sanger Institute Tree of Life Management, Samples and Laboratory team
- Members of Wellcome Sanger Institute Scientific Operations – Sequencing Operations
- Members of the Wellcome Sanger Institute Tree of Life Core Informatics team
- Members of the Tree of Life Core Informatics collective
- Members of the Project Psyche Community

### Wellcome Sanger Institute – Legal and Governance

The materials that have contributed to this genome note have been supplied by a Tree of Life collaborator. The Wellcome Sanger Institute employs a process whereby due diligence is carried out proportionate to the nature of the materials themselves, and the circumstances under which they have been/are to be collected and provided for use. The purpose of this is to address and mitigate any potential legal and/or ethical implications of receipt and use of the materials as part of the research project, and to ensure that in doing so, we align with best practice wherever possible. The overarching areas of consideration are:

- Ethical review of provenance and sourcing of the material
- Legality of collection, transfer and use (national and international).

Each transfer of samples is undertaken according to a Research Collaboration Agreement or Material Transfer Agreement entered into by the Tree of Life collaborator, Genome Research Limited (operating as the Wellcome Sanger Institute), and in some circumstances, other Tree of Life collaborators.
