# Supplementary material for: The genome sequence of the Pine Cone moth, Dioryctria mendacella (Staudinger, 1859) (Lepidoptera: Pyralidae)
Source: Wellcome Open Res. 2026 Sep 14;11:646. [Version 1] doi: 10.12688/wellcomeopenres.27800.1 (PMC13631444; doi:10.12688/wellcomeopenres.27800.1)
Supplement: Peer review history

# Reviewer report: Reviewer response for version 1

**DOI:** 10.21956/wellcomeopenres.30593.r168997

**Published:** 2026-10-01

**Authors:** Bengt Hansson

**Affiliations:** 1 Lund University, Lund, Sweden

This manuscript presents a high-quality genome assembly and annotation for *Dioryctria mendacella*, generated as part of Project Psyche. The authors report a chromosomal-scale assembly with the vast majority of the primary haplotype scaffolded into chromosome-level pseudomolecules, including the sex chromosomes, together with a mitochondrial genome assembly and gene annotation.

 The manuscript is clearly written and provides all essential assembly and annotation statistics. The methods are appropriate, the results are presented transparently, and the reported assembly metrics indicate a high-quality genomic resource that will be valuable for future research on Lepidoptera and comparative genomics.

Are the datasets clearly presented in a usable and accessible format, and the assembly and annotation available in an appropriate subject-specific repository?

Yes

Are sufficient details of the sequencing and extraction, software used, and materials provided to allow replication by others?

Yes

Are the rationale for sequencing the genome and the species significance clearly described?

Yes

Are the protocols appropriate and is the work technically sound?

Yes

Reviewer Expertise:

Evolutionary biology, population genetics, comparative genomics

I confirm that I have read this submission and believe that I have an appropriate level of expertise to confirm that it is of an acceptable scientific standard.

# Reviewer report: Reviewer response for version 1

**Published:** 2026-09-14

**Authors:** Automated Benchmarking

**Affiliations:** 1 Wellcome Sanger Institute, Hinxton, UK

As this article is a Genome Note, Automated Benchmarking has been used to determine whether the reported assembly passes three defined checks established by the Earth BioGenome Project: the raw and assembled data are publicly available, the Earth BioGenome Project three-part metric is met, and the BUSCO score passes the stated threshold. For Dioryctria mendacella (ilDioMend1), all three checks passed. Data available: pass. ENA study PRJEB82270, raw-read accessions ERR13946496 and ERR13947524, and assembly accessions GCA_964374295.1 and GCA_964374265.1 are public. Earth BioGenome Project three-part metric: pass. Haplotype 1 is reported as 7.C.Q65, meeting the 6.C.Q40 reference standard. BUSCO: pass. Haplotype 1, which is proposed as the reference assembly for this species, has 99.0% complete BUSCOs (98.6% single-copy and 0.4% duplicated). BUSCO software version: 5.5.0; lineage dataset: lepidoptera_odb10. It meets the target of greater than 90% complete and single-copy BUSCOs. This decision uses values reported in the Genome Note and public accession records.

Reviewer Expertise:

NA

I confirm that I have read this submission and believe that I have an appropriate level of expertise to confirm that it is of an acceptable scientific standard.
